# Early phase of shock formation in pair plasma colliding with electron–proton plasma

**DOI:** 10.1038/s41598-026-62899-0

**Published:** 2026-07-22

**Authors:** M. E. Dieckmann

**Affiliations:** Department of Science and Technology (ITN), Linköping University, Campus Norrköping, 60174 Norrköping, Sweden

**Keywords:** Astronomy and planetary science, Physics

## Abstract

We investigate the collisionless interaction between an electron–positron pair plasma and a magnetized electron–proton plasma using a three-dimensional particle-in-cell simulation. Our aim is to resolve the early stage of the formation of a discontinuity separating the inner cocoon formed by shocked pair plasma from the outer cocoon in relativistic jets and pair-plasma winds. An initially unmagnetized pair plasma impacts a background plasma permeated by a magnetic field perpendicular to the collision direction. The relative speed is 60% of the speed of light. The drift of electrons and positrons along the sharp, initially planar magnetic boundary destabilizes the boundary, fragmenting it into an ensemble of magnetic flux tubes. The magnetically reflected pair plasma then drives a strong, mildly relativistic shock mediated by the filamentation instability in front of the boundary. The heated and compressed pair plasma pushes the flux tubes deeper into the electron–proton plasma. The electric fields induced by the moving flux tubes and by the unequal densities of electrons and positrons behind the boundary accelerate ambient protons. The simulation resolves the intrinsic spatial and temporal scales governing flux-tube formation and pair shock evolution, providing quantitative guidance for laboratory laser–plasma experiments while directly informing models of astrophysical shocks.

## Introduction

Compact accreting objects (neutron stars and black holes) are often sources of relativistic outflows of electron-positron pair plasma. These outflows collide with the electrons, protons, and heavier ions of the ambient interstellar medium. Their interactions give rise to pulsar wind nebulae^1^ and relativistic collimated jets^2^.

The large scale of some outflows allows us to study their internal structure over a wide range of frequency bands. However, processes at the thin boundary between the pair plasma and the electron–proton plasma or at shocks can only be resolved in some cases^3^. The conversion of the directed flow energy into radiation near such a boundary can give rise to more energetic and variable radiation than that emitted from the surrounding flow providing some insight into the plasma dynamics at the boundary. Laboratory experiments and simulations can complement astrophysical observations by providing additional insight.

The low number density of the pair plasma and the ionized interstellar medium implies that binary interactions (Coulomb collisions) between charged particles rarely occur on the time scales of interest. Charged particles in a collisionless plasma interact via the macroscopic electromagnetic field. These electromagnetic fields are induced by the electric current which is tied to the collective motion of a large number of charged particles.

State-of-the-art lasers reach intensities and energies, which can create electron-positron pairs to enable collisionless collective interactions between the charged particles^4^. At present, collisionless processes that can be studied in laboratory experiments are limited to wave instabilities^5^; electromagnetic waves grow at the expense of the drift velocity the pair plasma has relative to ambient plasma. The ambient plasma can, for example, be ionized residual gas.

Collisionless processes in an electron-positron-ion plasma can be numerically modeled with particle-in-cell (PIC) simulation codes^6,7^. These codes are computationally demanding, especially if they resolve three spatial dimensions. If run on massively parallel computers, they can nevertheless resolve spatial and time scales outside reach for laboratory experiments. PIC simulations can reveal the scales on which collisionless shocks and discontinuities form, and suitable plasma conditions, providing important insight for future experimental studies.

The present simulation is not intended to model a specific astrophysical object or laboratory experiment. Instead, it investigates generic collisionless processes that arise when a relativistic electron-positron plasma encounters a magnetized electron–proton plasma. Such interactions are relevant to relativistic astrophysical outflows, including pulsar winds and jets, while also being accessible in scaled laboratory experiments. Although present-day PIC simulations cannot reproduce the full range of parameters encountered in astrophysical systems, the spatial and temporal scales resolved by large three-dimensional simulations are comparable to those accessible in contemporary laser-plasma experiments. The simulation therefore serves as a bridge between astrophysical scenarios and laboratory studies by identifying the kinetic processes and characteristic scales that govern the formation of magnetic structures, shocks, and energy transfer between pair and electron–proton plasmas.

Here we model with a three-dimensional PIC simulation the collision of a pair plasma and an electron–proton plasma at speed 0.6*c*. This study expands on a previous three-dimensional PIC simulation study that focused on the structure of the discontinuity that separates both plasmas^8^. The initial and boundary conditions of this new simulation led to the self-consistent formation of an inner cocoon between the collisionless shock in the pair plasma and the discontinuity. A drift instability^9^ between the hot pair plasma and the initially planar magnetic boundary let it change into a transition layer of magnetic flux tubes. We also observe bulk acceleration of protons to a speed that exceeds by far the fast magnetosonic speed in the electron–proton plasma.

Our paper is structured as follows. The section “Simulation code and setup” discusses the simulation code and setup. The section “Results” presents the results, which are summarized in the section “Discussion”.

## Simulation code and setup

The PIC simulation code *EPOCH*^7^ places the electric field $\textbf{E}$, the magnetic field $\textbf{B}$, and the macroscopic current density $\textbf{J}$ on a numerical grid. We use uniform cell spacing $\Delta _s$ in all directions. The current density $\textbf{J}$ updates $\textbf{E}$ and $\textbf{B}$ through the laws of Ampère and Faraday. Esirkepov’s scheme^10^, on which *EPOCH* is based, fulfills Gauss’ law and the magnetic divergence law $\nabla \cdot \textbf{B}=0$ to round-off precision.

Our simulation represents electrons (e), positrons (p) and protons (i) with the proton-to-electron mass ratio $m_i/m_e = 1836$. Each plasma species is resolved by an ensemble of computational particles (CP’s) with a charge-to-mass ratio that equals that of the represented particle.

One computational cycle covers a time step $\Delta _t$. In each cycle, $\textbf{E}$ and $\textbf{B}$ are interpolated to the position of each CP and update its momentum using the relativistic Lorentz force equation. The current each CP carries is interpolated to the grid. A summation of all interpolated currents gives $\textbf{J}$, which updates $\textbf{E}$ and $\textbf{B}$.

**Fig. 1:**
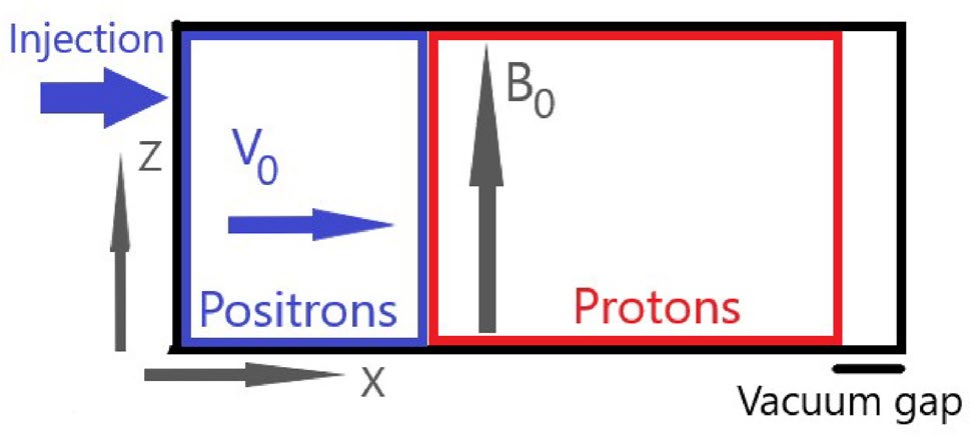
Simulation setup of the 3D simulation box (y-axis points into the plane). The red domain is filled with an electron–proton (ei) plasma at rest and separated by a vacuum gap from the right open boundary. The blue domain is filled with an electron-positron (ep) plasma with the mean speed $\textbf{v}_0=(v_0,0,0)$. An ep plasma is continuously injected at the open left boundary. A background magnetic field $\textbf{B}_0$ pointing to increasing *z* fills the red domain.

At time $t=0$, the three-dimensional simulation box is subdivided into three domains as shown in Fig. 1.

The red domain, which represents a stellar wind or interstellar medium, is filled with electrons with temperature $T_e=4$ keV and protons with temperature $T_i=T_e/2$. An electron or positron temperature of 4 keV corresponds to a thermal energy $k_BT_e/(m_e c^2) \approx 7.8 \times 10^{-3}$ ($k_B$: Boltzmann constant). We refer to the electron–proton plasma as the ei plasma. It is separated by a vacuum gap from the open boundary.

The temperatures of the ei plasma exceed those of the stellar wind or interstellar medium. Hotter electrons allow us to use larger cell sizes $\Delta _s$ and time steps $\Delta _t$. During the simulation, particles of both species will be accelerated to much larger kinetic energies, which will make the results less dependent on the initial temperatures.

Both species have a number density $n_0$. The plasma frequency of electrons is $\omega _{pe}={(e^2n_0/m_e\epsilon _0)}^{1/2}$, where *e* is the elementary charge and $\epsilon _0$ the vacuum permittivity. The ei plasma is permeated by a spatially uniform magnetic field $\textbf{B}_0=(0,0,B_0)$, which has an electron gyro-frequency $\omega _{ce}=eB_0/m_e$ with $\omega _{ce}/\omega _{pe}=0.089$. The plasma $\beta = 2\mu _0 n_0k_BT_e / B_0^2$ with respect to the thermal pressure of the electrons is about 2, where $\mu _0$ the vacuum permeability.

The blue domain is filled with an unmagnetized electron-positron (ep) plasma. Electrons and positrons both have the number density $n_0$ in the rest frame of the simulation box. Their temperature is $T_e$ and their mean velocity vector $\textbf{v}_0=(v_0,0,0)$ with $v_0=0.6$c. A charge and current neutral ep plasma with the same density, mean velocity, and temperature is injected at the left boundary $x=0$ in Fig. 1.

Length scales are expressed in units of the electron skin depth $\lambda _e = c/\omega _{pe}$. The kinetic equations can be specified in normalized units. A larger value of the plasma density $n_0$ leads to smaller structures but the physics remains unchanged. We set the particle number density in our simulation to $n_0=400\, \textrm{cm}^{-3}$, which gives $\lambda _e \approx 266$ m. The length of the box $L_x=507.6\lambda _e$ along *x* is resolved by 3600 grid cells. The lengths $L_y=59.2\lambda _e$ and $L_z=56.4\lambda _e$ along *y* and *z* are resolved by 420 and 400 grid cells, respectively. The boundary conditions are open along *x* and periodic along the other directions. We employ 8 CPs per cell for each plasma species and the pair plasma, which is injected at $x_{min}=0$.

At time $t=0$, the boundary between the blue and red domains is located at $x=141\lambda _e$. The vacuum gap covers the range $479.4\lambda _e \le x \le 507.6\lambda _e$. The simulation time $t_{sim} = 3100 \, \omega _{pe}^{-1}$ is covered by $4 \times 10^4$ time steps $\Delta _t$.

Volume data is averaged over blocks with side length 2 cells in all directions. The data labeled “box-averaged data” was averaged in all *y* and *z*. The number densities are given in units of $n_0$. Magnetic pressure $P_B = \textbf{B}^2 / 2 \mu _0$ is expressed in units of electron thermal pressure $P_{the}=n_0 k_B T_e$ giving $P_B = \beta ^{-1}$. At $t=0$, $P_B=0.5$ in the red domain, and 0 outside it.

The characteristic speeds in the ep plasma are the electron thermal speed $v_{te}$, which is also that of the positrons, and the sound speed $c_{se}$. In the ei plasma, we have the ion acoustic speed $c_{si}$ with the values $\gamma _e = 5/3$ and $\gamma _i=3$ for the adiabatic constants, the Alfvén speed $v_A$, and the fast magnetosonic speed $v_{fm}$. Table 1 lists their definitions and values in the plasma in the blue and red domains.

**Table 1:** The definition and value for each characteristic speed in the blue domain (upper two rows) and red domain.

Definition	Value
$v_{te}=(k_BT_e/m_e)^{1/2}$	2.7$\times 10^7$m/s
$c_{se}=(3k_B T_e/m_e)^{1/2}$	4.6$\times 10^7$m/s
$c_{si}=(k_B(\gamma _eT_e+\gamma _iT_i)/m_i)^{1/2}$	9.8$\times 10^5$m/s
$v_A = B_0/(\mu _0 m_i n_0)^{1/2}$	6.2$\times 10^5$m/s
$v_{fm}=(v_A^2+c_{si}^2)^{1/2}$	1.2$\times 10^6$m/s

## Results

### Time evolution

**Fig. 2:**
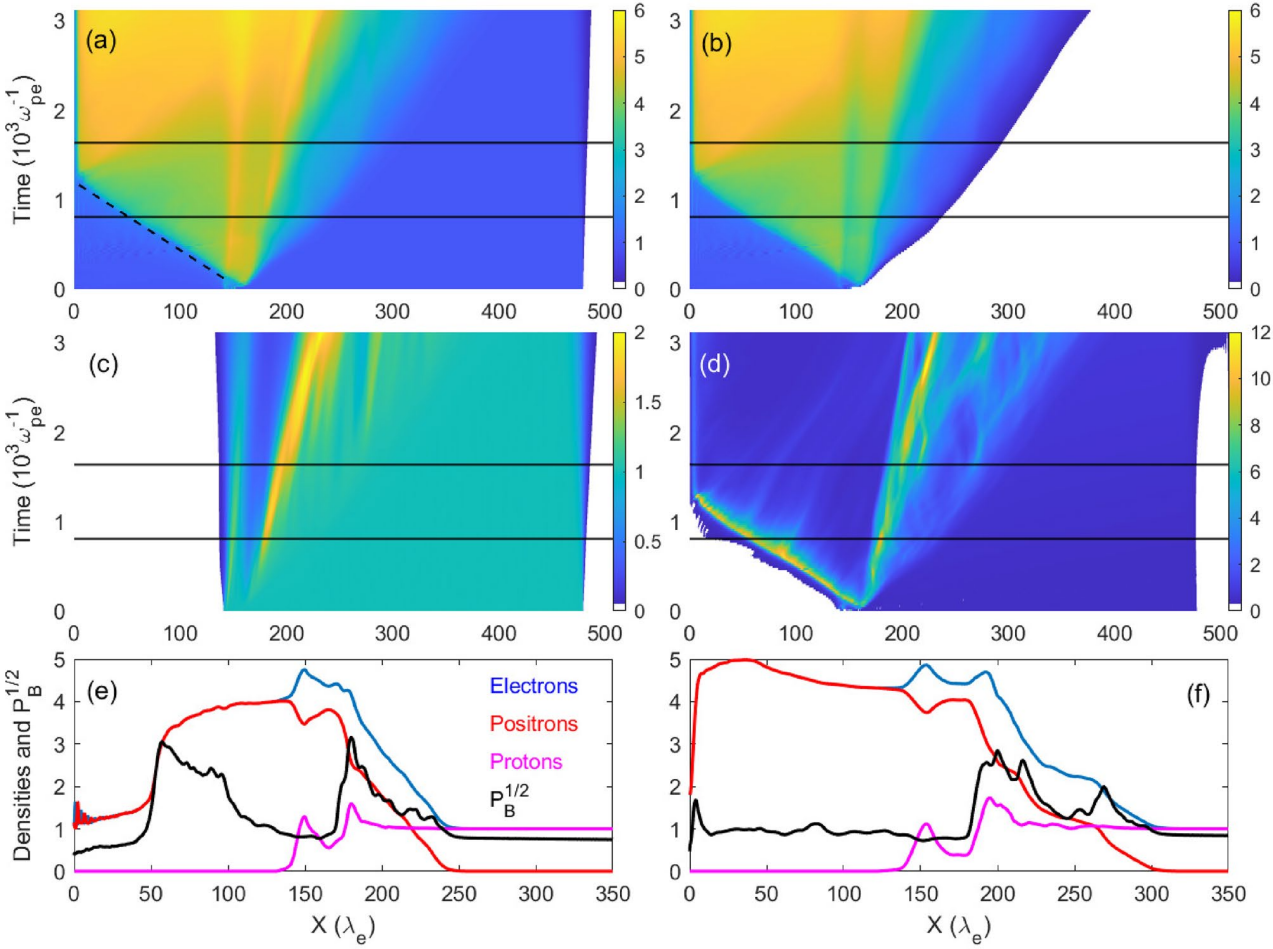
Box-averaged number densities and magnetic pressure. The solid horizontal lines in (**a**–**d**) correspond to the times $807 \omega _{pe}^{-1}$ and $1614\omega _{pe}^{-1}$. The electron density is shown in (**a**). The dashed line marks the speed $-0.13c$ in the rest frame of the simulation box. The densities of the positrons and protons are shown in (**b**) and (**c**), respectively. (**d**) Magnetic pressure $P_B$. Panels (**e**, **f**) show the distributions in (**a**–**d**) at the times corresponding to the solid lines at $t=807\omega _{pe}^{-1}$ and $t=1614\omega _{pe}^{-1}$, respectively. Both panels show $P_B^{1/2}$. The color legend in (**e**) applies also to (**f**).

Figure 2 shows the box-averaged number densities $n_e(x,t)$ of electrons, $n_p(x,t)$ of positrons, $n_i(x,t)$ of protons and $P_B(x,t)$.

At low *x* and *t*, the electrons in Fig. 2a have a density $\approx 1$. The ep plasma is reflected near the boundary between the blue and red domains at $x=141\lambda _e$, creating a diamagnetic electric current that pushes the magnetic field in the direction of $\textbf{v}_0$.

A wave with density $\approx 4$ and speed -0.13c propagates back to the injection boundary at $x=0$ reaching it at $t\approx 1100\omega _{pe}^{-1}$. Its front leaves the simulation box. A new wave with density 5 is launched at $x=0$ and propagates to increasing *x* at a speed $\sim$ 0.15c. The excess density $\approx 1$ corresponds to that injected at the boundary at $x=0$.

The open boundary at $x=0$ allows some of the shock-heated electrons and positrons to escape. The observed increase of the pair plasma density near the boundary at $x=0$ shows that hardly any pair plasma escapes. This is probably caused by the momentum transfer from the injected pair plasma beam to the pair plasma, which is already in the simulation box, which keeps the latter separated from the open boundary.

The propagation speed $\sim$0.15c of the density wave equals the sound speed $c_{se}$ in the pair plasma. However, $c_{se}$ was calculated for $T_e$, which is below the temperature of the shock-heated electrons and the exact correspondence might be a coincidence. The density wave reaches the initial location of the boundary $x=141\lambda _e$ at $t\approx 2000\omega _{pe}^{-1}$. Figure 10 shows that the pair plasma has an approximately thermal distribution when it arrives at the electron–proton plasma.

This injector artifact increases the thermal pressure of the pair plasma by about 20% but is unlikely to introduce additional instabilities. Its effects will be limited to $x\le 220 \lambda _e$, which is the position reached by the density wave at $t=3000\omega _{pe}^{-1}$ in Fig. 2a. We will later see that the plasma evolution is qualitatively unchanged by the arrival of this artifact wave, which evidences the robustness of our results against moderate changes in the thermal pressure of the pair plasma. Such changes are likely to occur also in astrophysical settings.

The front of the electrons, which expands into the red domain, reaches $x\approx 400\lambda _e$ at time $t_{sim}$. Two density peaks are visible in Fig. 2a. The one at $x\approx 150\lambda _e$ moves at the speed $\approx 2 \times 10^6$m/s and the one at $x\approx 200\lambda _e$ at almost 3 times that speed. These peaks are most pronounced at $t\approx 1614\omega _{pe}^{-1}$, which can be seen in Fig. 2f for the electrons and protons. Electrons expand into the vacuum gap, but do not reach the open boundary at $x=507.6 \lambda _e$ in significant numbers, ensuring charge conservation.

At $x<140\lambda _e$, the positron distribution shown in Fig. 2b is identical to that of the electrons. It does not show the narrow density peaks in the interval $140\lambda _e \le x \le 210 \lambda _e$ the electron density had. During time $t_{sim}$, the front of the positrons expands from $x = 140 \lambda _e$ to $x\approx 370 \lambda _e$ giving the average speed 0.075*c*, which is well below $v_0$ and close to $v_{te}$; the injected ep plasma loses its directed flow energy to the magnetized ei plasma.

Figure 2c shows the proton density. Its thermal speed and thermal pressure allow it to expand to $x\le 140 \lambda _e$ and into the vacuum gap. Two density peaks are located near $x\approx 150 \lambda _e$ and $x\approx 200 \lambda _e$. They coincide with the pronounced peaks in the electron distribution; electrons shield the proton charge while positrons are repelled by it. At the end of the simulation at $t_{sim}$, the proton density is visibly modulated to about $x\approx 300\lambda _e$. Visibly modulated means here that it is not constant and uniform, which would be the case if the protons still had their initial density.

Figure 2d reveals that one strong peak in the magnetic pressure coincides with the wave front in the ep plasma denoted by the dashed line in Fig. 2(a). Another strong peak is located near the fast-moving proton density wave near $x=200\lambda _e$.

Figure 2e shows the distributions of (a-d) at the time $807 \omega _{pe}^{-1}$ before the ep density wave in the blue domain crossed the open boundary at $x=0$. The matching distributions of the electron density for $x<141 \lambda _e$ imply that the plasma is quasi-neutral. Both densities rise from a value $\approx 1$ at $x\approx 40\lambda _e$ to 4 at $x\approx 100\lambda _e$. At $x\approx 55\lambda _e$, the magnetic pressure reaches a peak value of 9 and remains above 5 until $x\approx 100 \lambda _e$. The magnetic pressure decreases at larger *x* and reaches its minimum near $x=150\lambda _e$, where the proton density starts to rise. It passes through another peak with the value $P_{B}\approx 9$ near $x\approx 180 \lambda _e$ where the proton density has a second peak. It decreases for higher *x* and converges to its initial value in the red domain at $x\approx 250\lambda _e$. This location coincides with the front of the positron density.

Figure 2f shows the distributions at time $1614 \omega _{pe}^{-1}$. The ep plasma reaches the density value 5 at $x\approx 40\lambda _e$. At this time, the injector is immersed in the downstream plasma of the shock that has escaped through the boundary. Electrons and positrons are injected with density 1 each, which adds to the density 4 of the downstream plasma and its thermal pressure.

Once the ep plasma reaches the first proton density peak at $x\approx 150 \lambda _e$, the electron and positron densities no longer match. The mismatch is caused by the proton charge and the need to maintain quasi-neutrality. This proton peak is located close to the initial boundary at $x=141\lambda _e$ and is formed by protons that were not accelerated much before the magnetic field was expelled. A Hall electric field, which accelerates the protons, requires a drift current perpendicular to the magnetic field. This drift current does not develop instantly. It is likely that these protons are located in an interval that was demagnetized before the drift current grew. The value of $P_{B}$ is practically constant near this first peak of the proton density. If protons are still accelerated at this position, the acceleration can not be caused by an electric field associated with a magnetic pressure gradient. The densities of the electrons and positrons start diverging at the original boundary $x=141\lambda _e$ in Figs. 2e, f.

The proton density distribution and the divergence of the electron- and positron density distributions are closely correlated. The latter is thus caused by the need to fulfill quasi-neutrality. A mixing of the electrons of the pair cloud and of the electron–proton plasma results in the rise of the electron density. The electric current associated with the moving denser electrons cannot be balanced by the positron current. An electric field is induced by the net current, which slows down the electrons and accelerates the positrons and protons, providing an effective means to transfer the momentum of the pair cloud to that of the protons. The magnetic pressure increases to a value of 9 near the second peak of proton density at $x\approx 190 \lambda _e$ and decreases to its initial value at the front of the positron cloud.

In what follows, we examine the multidimensional density and magnetic pressure distributions to gain additional insight into the observed structures.

### Time $t=86\omega _{pe}^{-1}$

**Fig. 3:**
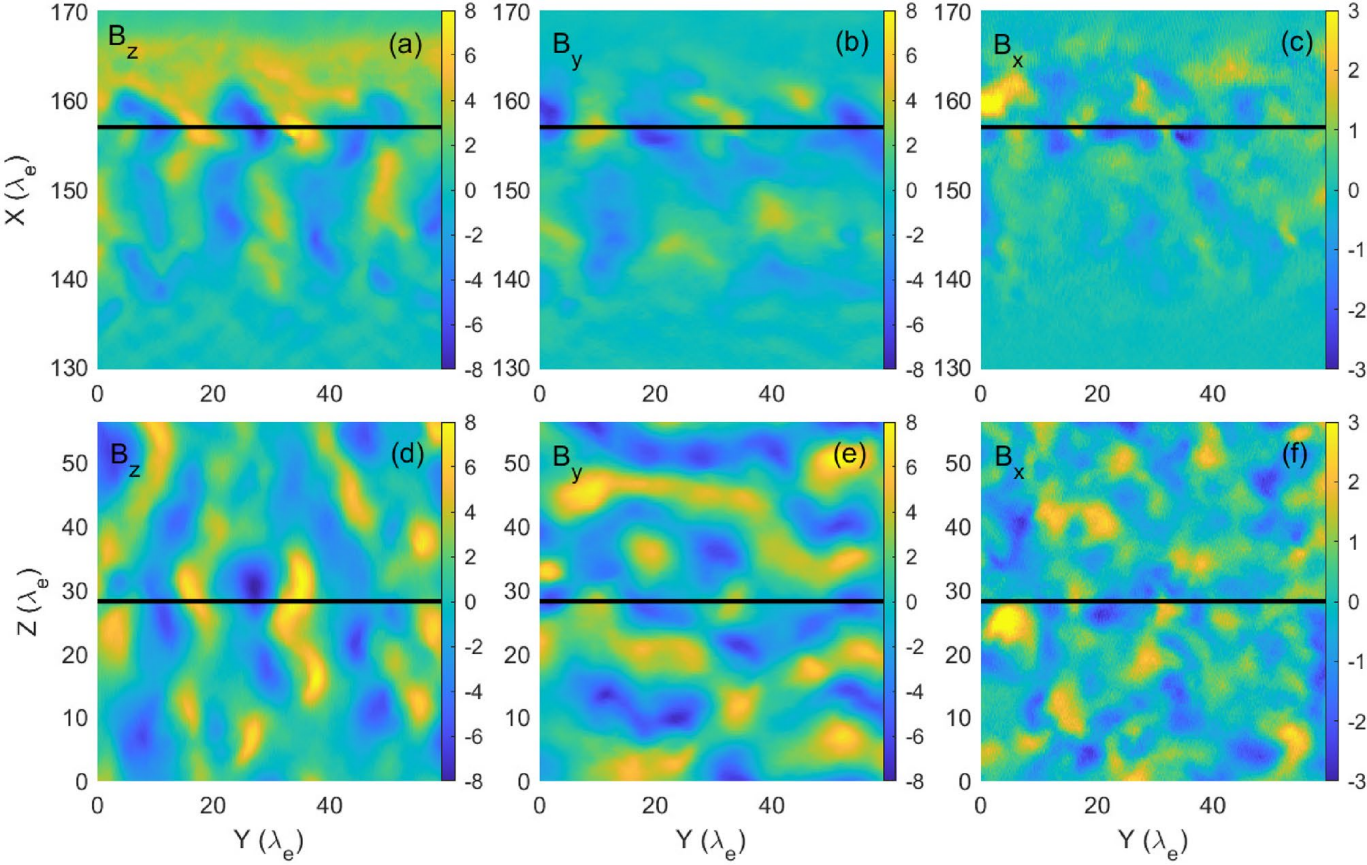
The magnetic $B_x$, $B_y$, and $B_z$ components at time $86\omega _{pe}^{-1}$ near the collision boundary $x=141\lambda _e$ between the ep plasma and the ei plasma. The magnetic fields are expressed in units of $B_0$. The upper row shows them along the slice $z=28.4 \lambda _e$. The lower row shows the fields in the plane defined by $x=157\lambda _e$. The black lines show the positions of the orthogonal slice plane.

Our initial conditions do not depend on *y* and *z*. Figure 3 demonstrates, however, that the magnetic amplitudes vary in all directions as early as $t=86\omega _{pe}^{-1}$; a collisionless instability with a high exponential growth rate destabilized the magnetic boundary.

Figure 3a shows that $B_z(x,y)$ is spatially uniform and close to $B_0$ for $x\gtrsim 167\lambda _e$. The magnetic field in this interval has not yet been compressed or deformed by the ep plasma. The magnetic amplitude increases to about $5B_0$ and varies in strength along *y* in the interval $162\lambda _e \le x \le 167\lambda _e$. It oscillates along *y* near $x=157\lambda _e$ with an amplitude close to $8B_0$ forming what seems to be a magnetowave. Oscillations with the same wavelength and a lower amplitude persist to $x\approx 145\lambda _e$.

Figure 3b shows oscillations of $B_y(x,y)$ along *y* at $x\approx 157\lambda _e$. Their correlation with $B_z(x,y)$ suggests that $B_z(x,y)$ drives the oscillations, while $B_y(x,y)$ maintains $\nabla \cdot \textbf{B}=0$. Figures 3d, e show that $B_y(y,z)$ and $B_z(y,z)$ oscillate also along *z*. The filaments in $B_z(y,z)$ are on average aligned with *z*, while those in $B_y(y,z)$ are aligned with *y*; the magnetic field tends to be rotational in the y-z plane. Figures 3c, f show that $B_x(x,y)$ and $B_x(y,z)$ oscillate with lower amplitude and are not strongly correlated with the other magnetic components.

**Fig. 4:**
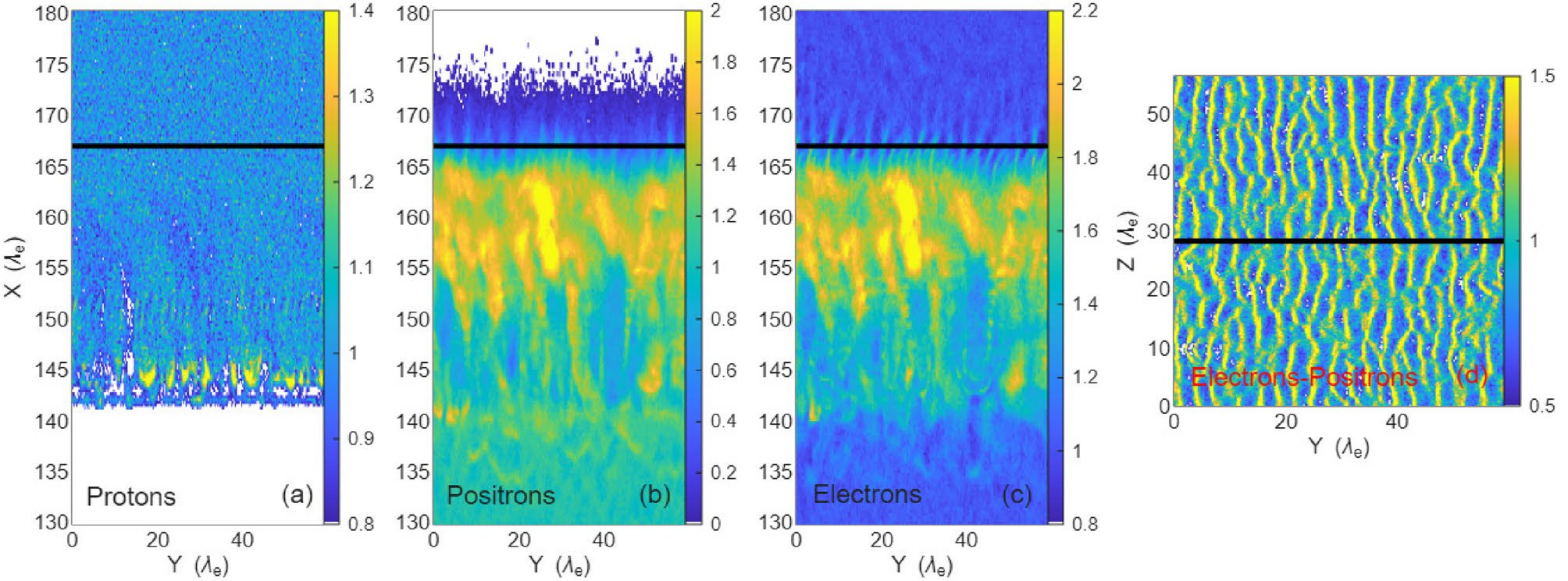
Plasma density distributions at $t=86\omega _{pe}^{-1}$. Panel (**a**) shows the proton distribution at $z=28.4\lambda _e$. Panels (**b**, **c**) show the distributions of positrons and electrons in the same spatial interval and at $z=28.4\lambda _e$. The horizontal lines in panels (**a**–**c**) show $x=167\lambda _e$. Panel (**d**) shows the density difference $n_e-n_p$, which is proportional to the negative charge density, in the plane perpendicular to *x*. The horizontal line shows $z=28.4\lambda _e$.

Figure 4 examines the distributions of the three plasma species near $x=167\lambda _e$, where the magnetic field became perturbed.

Figure 4a shows that the mean value and statistical fluctuations of the proton density are spatially uniform near $x=167\lambda _e$. At this location, the protons did not yet react to electromagnetic fields. Modulations of the proton density appear in the interval $142\lambda _e< x < 162\lambda _e$. The densities of positrons and electrons in Figs. 4b, c show filaments near $x=167\lambda _e$, which are aligned on average with *x* and are separated by $\sim 3\lambda _e$ along *y*. Filaments in both plasma species are in phase and those of the electrons are more pronounced. Both densities show large-amplitude fluctuations in $150\lambda _e \le x \le 165 \lambda _e$. Figure 4d shows the difference between both densities in the plane $x=167\lambda _e$. This difference is proportional to the negative charge density. Charge density waves with a wave vector along *y* and a wavelength $3\lambda _e$ fill the displayed y-z plane.

Our simulation does not distinguish between the electrons, which were originally located in the red domain in Fig. 1, and those of the pair plasma. However, at least the positrons in Fig. 4b extend well into the interval with a homogeneous $\textbf{B}_0$. These positrons gyrate in $\textbf{B}_0 \parallel z$ and will have a high velocity along *y*.

Electrons drifting in a magnetic field with an amplitude that does not vary with *y* lead to an electron-cyclotron drift instability^11^, which allows charge density waves with a short wavelength to grow that propagate in the drift direction. Figure 4d shows that drifting electrons and positrons also lead to instability.

This instability breaks the spatial uniformity of the plasma perpendicular to *x*. Figures 4b, c also reveal that the front of the ep plasma oscillates on a larger scale. The color contour with density 1.2 touches the line $x=167\lambda _e$ near $y=25\lambda _e$, while it is located at $x\approx 166\lambda _e$ near $y=18\lambda _e$ and $y=42\lambda _e$.

### Time $t=807\omega _{pe}^{-1}$

**Fig. 5:**
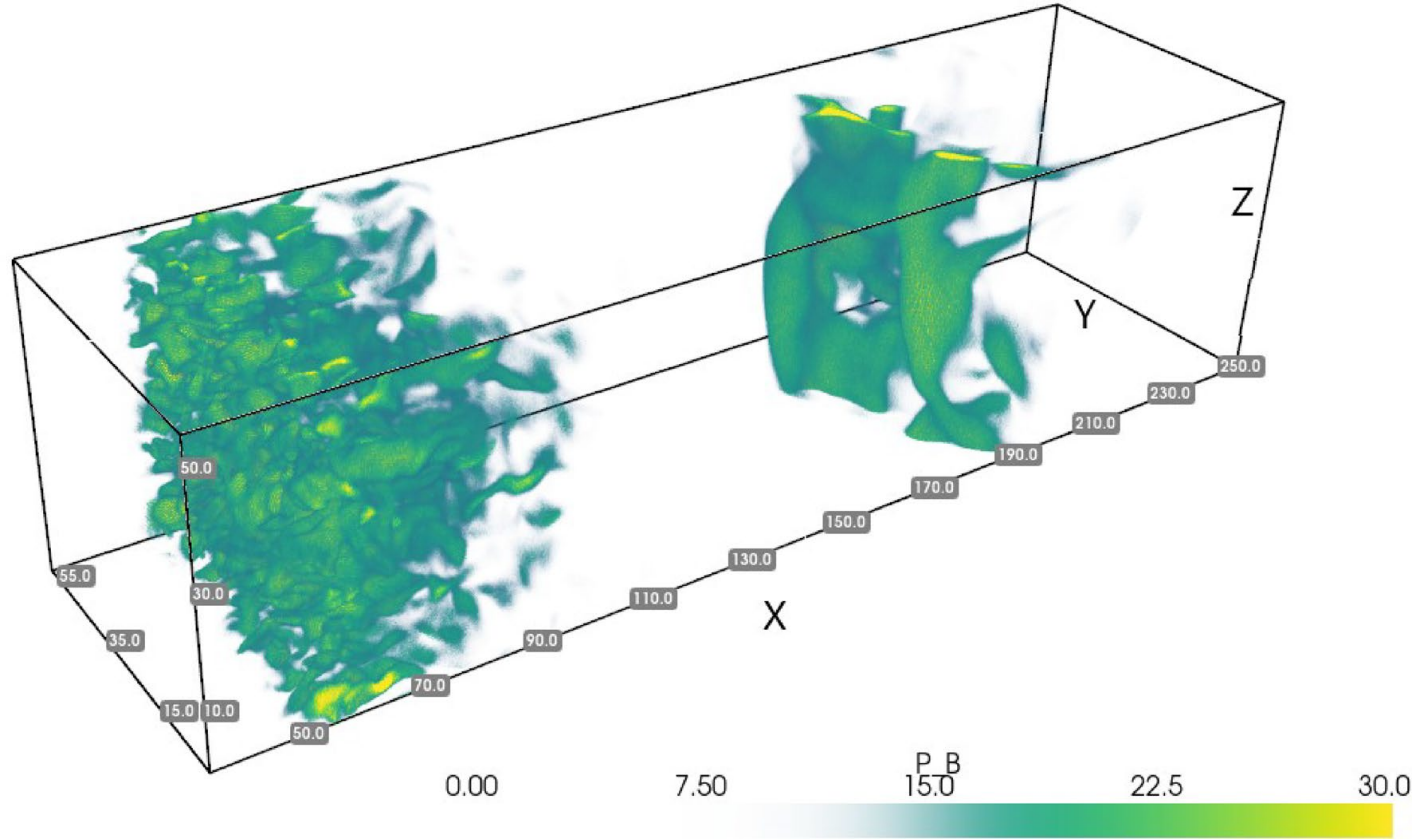
The three-dimensional distribution of the magnetic pressure $P_B$ at time $807\omega _{pe}^{-1}$. The color scale is clamped to the maximum value 30. The magnetic structure between $x=50\lambda _e$ and $x=110\lambda _e$ is the transition layer of an ep plasma shock. The magnetic structure between $x=190\lambda _e$ and $x=230\lambda _e$ is the initially uniform $\textbf{B}_0 \parallel z$, which was piled up by the ep plasma and changed into magnetic flux tubes that reach $P_B$ values exceeding 40.

Figure 5 shows $P_B(x,y,z)$ at time $t = 807\omega _{pe}^{-1}$. The magnetic field is no longer spatially uniform along *y* and *z* in $170\lambda _e \le x \le 210\lambda _e$.

It consists of flux tubes with a preferential alignment with *z* and a diameter of about 10$\lambda _e$. Given that the wavelength of the charge density waves observed before was about $3\lambda _e$, the surface of the flux tubes may no longer be planar enough to facilitate this instability, which would stabilize the flux tubes.

Magnetic structures are also present in the interval $50\lambda _e \le x \le 110\lambda _e$. At $t=0$, the magnetic field amplitude was zero in this interval, and the fields grew from noise levels. These magnetic structures have a preferential alignment with the x-axis and a diameter of a few $\lambda _e$, which is indicative of a filamentation instability also called beam-Weibel instability. It mediates high Mach number shocks in ep plasma.

The growth of these long-lived magnetic structures, and how they mediate the shock transition layer in a collisionless mildly relativistic shock in pair plasma, was observed and discussed in^12^. The shock formation was modelled with a PIC simulation in two spatial dimensions. These simulations can resolve the growth and saturation of the filamentation instability, but the absent third dimension implies that they cannot resolve correctly the long-term evolution of the shock.

According to Fig. 2a, the shock propagates at speed of -0.13c in the box frame or 0.68c in the rest frame of the injected ep plasma. Its speed $\approx 4c_{se}$ makes it a strong shock. The density jump in a strong MHD shock with a Mach number 4 is about 3^13^, which explains the density change from 1 to 4 observed in Fig. 2e.

**Fig. 6:**
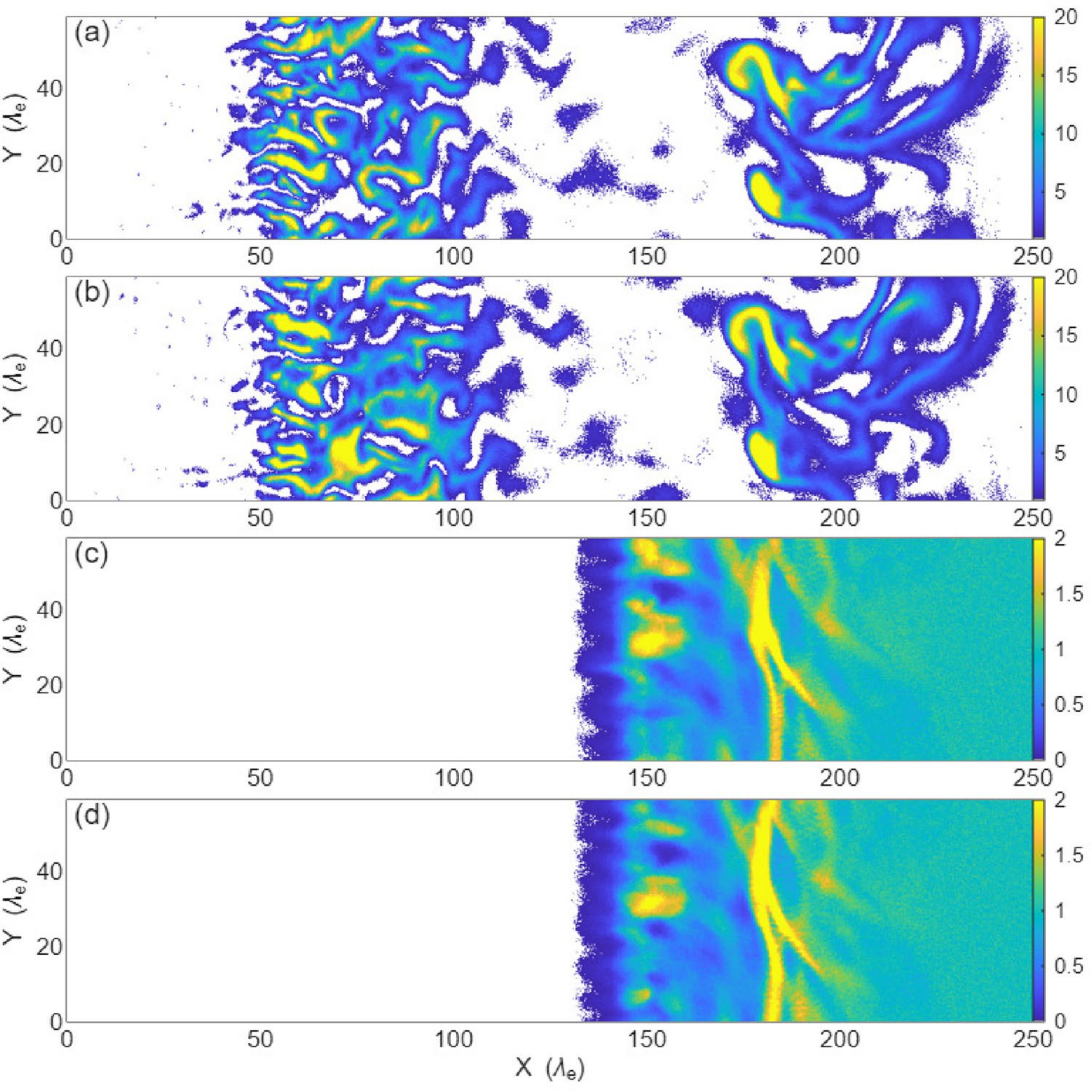
Slices of the magnetic pressure and proton density at time 807$\omega _{pe}^{-1}$. Panel (**a**) shows $P_B$ at $z = 28.2\lambda _e$ and (**b**) at $32.4 \lambda _e$. Values below 1 are set to white and the color scale is clamped at the maximum value 20. Panels (**c**, **d**) show the proton densities at $z = 28.2\lambda _e$ and $z=32.4\lambda _e$, respectively.

Figure 6 compares the proton density and the magnetic pressure $P_B(x,y,z)$ in the slices $z=28.2\lambda _e$ and $32.4\lambda _e$. The displayed domain along *x* and *y* is identical to that in Fig. 5 but we place the tick marks at different positions on both axes.

Figures 6a, c and  6b, d confirms that the shock transition layer in $50\lambda _e \le x \le 110 \lambda _e$ is immersed in the ep plasma and detached from the boundary of the blue domain $x=141\lambda _e$. The magnetic field is weak in the interval between $x=135\lambda _e$ and $170\lambda _e$. Two strong magnetic field structures are present at $x\approx 180\lambda _e$. They are separated along *y* by the distance $\approx L_y/2$. This wavelength is equal to that of the long oscillation of the ep plasma front along *y* near $x\approx 167\lambda _e$ in Fig. 4b, c, suggesting that both are connected. The magnetic structures in Fig. 6(a, b) are the cross sections of the flux tubes visualized in Fig. 5.

The ep plasma expands into the region filled with protons and a perpendicular magnetic field. Once the instability has broken up the initially homogeneous magnetic field into flux tubes, the ep plasma can expand deeper into the ei plasma and rearrange the magnetic field further ahead. A transition layer develops, which consists of strong and nonuniform magnetic field structures. The magnetic flux tubes in Fig. 6a, b have magnetic pressures that reach values of $P_B(x,y)=20$ and above. They move slowly enough to accelerate protons and modulate the proton density distributions in Figs. 6c, d effectively coupling energy from the ep plasma to the protons. The likely mechanism that accelerates the protons is the electric field, which is caused by the transport of trapped electrons and positrons by the magnetic field. Their densities are unequal, which results in a net current that induces an electric field.

### Intermediate time $t=1614\omega _{pe}^{-1}$

**Fig. 7:**
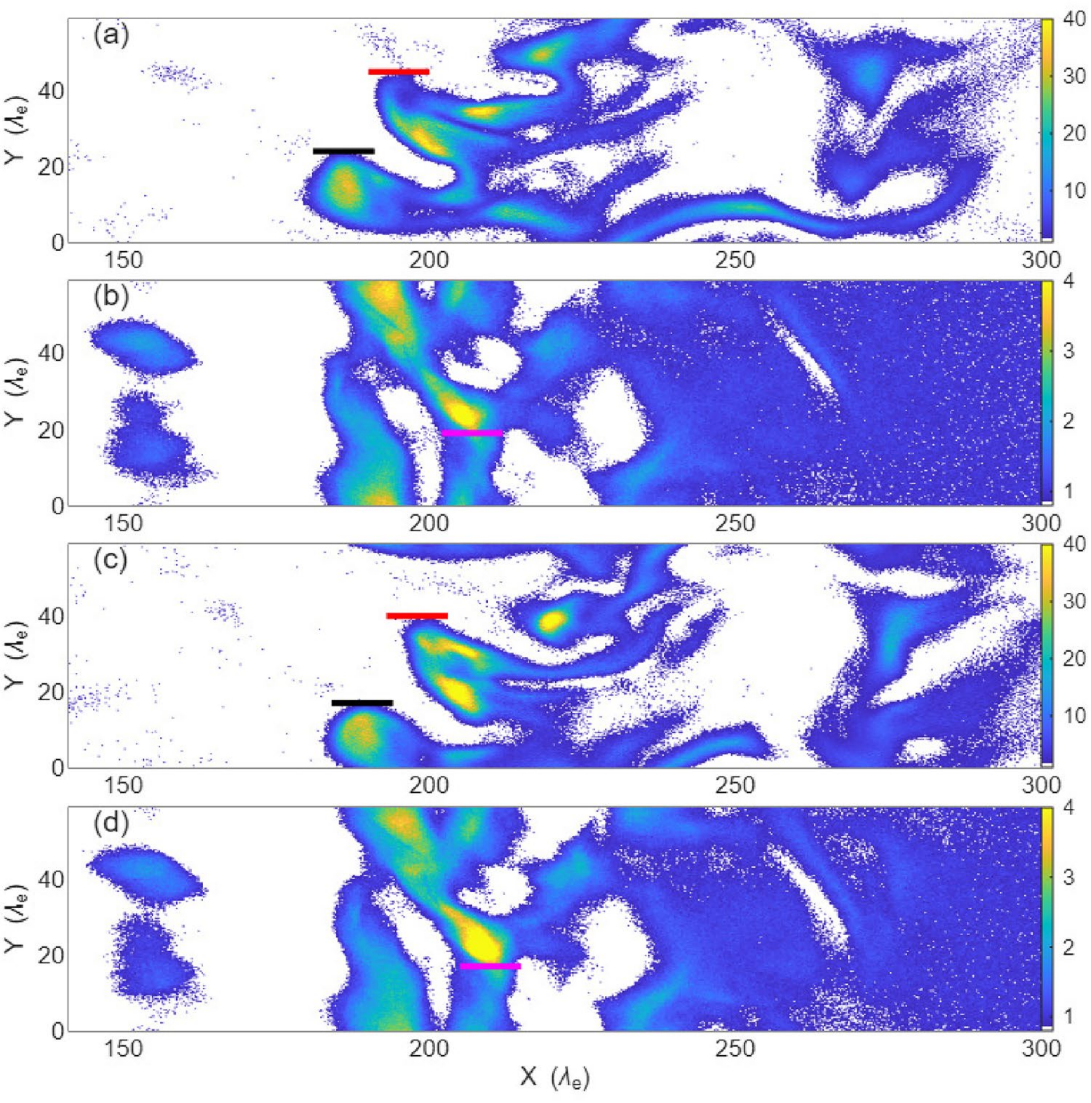
Magnetic pressure $P_B$ and proton density. (**a**) $P_B$ at time $1614\omega _{pe}^{-1}$. The black line is centered at $(x,y)=(186\lambda _e,24\lambda _e)$ and the red line at $(195\lambda _e,45\lambda _e)$. (**b**) shows the proton density at the same time. The magenta line is centered at $(206\lambda _e,19\lambda _e)$. (**c**, **d**) show $P_B$ and the proton density at time $1718\omega _{pe}^{-1}$. The black and red lines in (c) are centered at $(189\lambda _e,17\lambda _e)$ and $(198\lambda _e,40\lambda _e)$, respectively. The magenta line in (d) is centered at $(210\lambda _e,17\lambda _e)$.

Figures 7a, b show $P_B(x,y)$ and the proton density at $z=14.1\lambda _e$ and time $t=1614\omega _{pe}^{-1}$. Figure 7c, d show both at the same value of *z* and $1718\omega _{pe}^{-1}$. Movie 1 animates both data sets over time.

We can estimate the propagation speed of some magnetic and proton density structures from the black, red, and magenta lines plotted in Fig. 7. During the time interval $\delta _t= 104\omega _{pe}^{-1}$, the center of the black and red lines in Figs. 7a, c have been displaced by the vectors $(3\lambda _e,-7\lambda _e)$ and $(3\lambda _e,-5\lambda _e)$, respectively. Their speeds along *x* and *y* are about $5\times 10^6$ m/s and $-10^7$m/s, respectively. The proton structure in Fig. 7b, d moved $(3\lambda _e,-3\lambda _e)$, giving it the speed $5\times 10^6$m/s along *x* and $-5\times 10^6$m/s along *y*. These speeds are higher than $v_{fm}$, below $c_{se}$ and comparable to the speed of the high density structure in the box-averaged proton density in Fig. 2c.

**Fig. 8:**
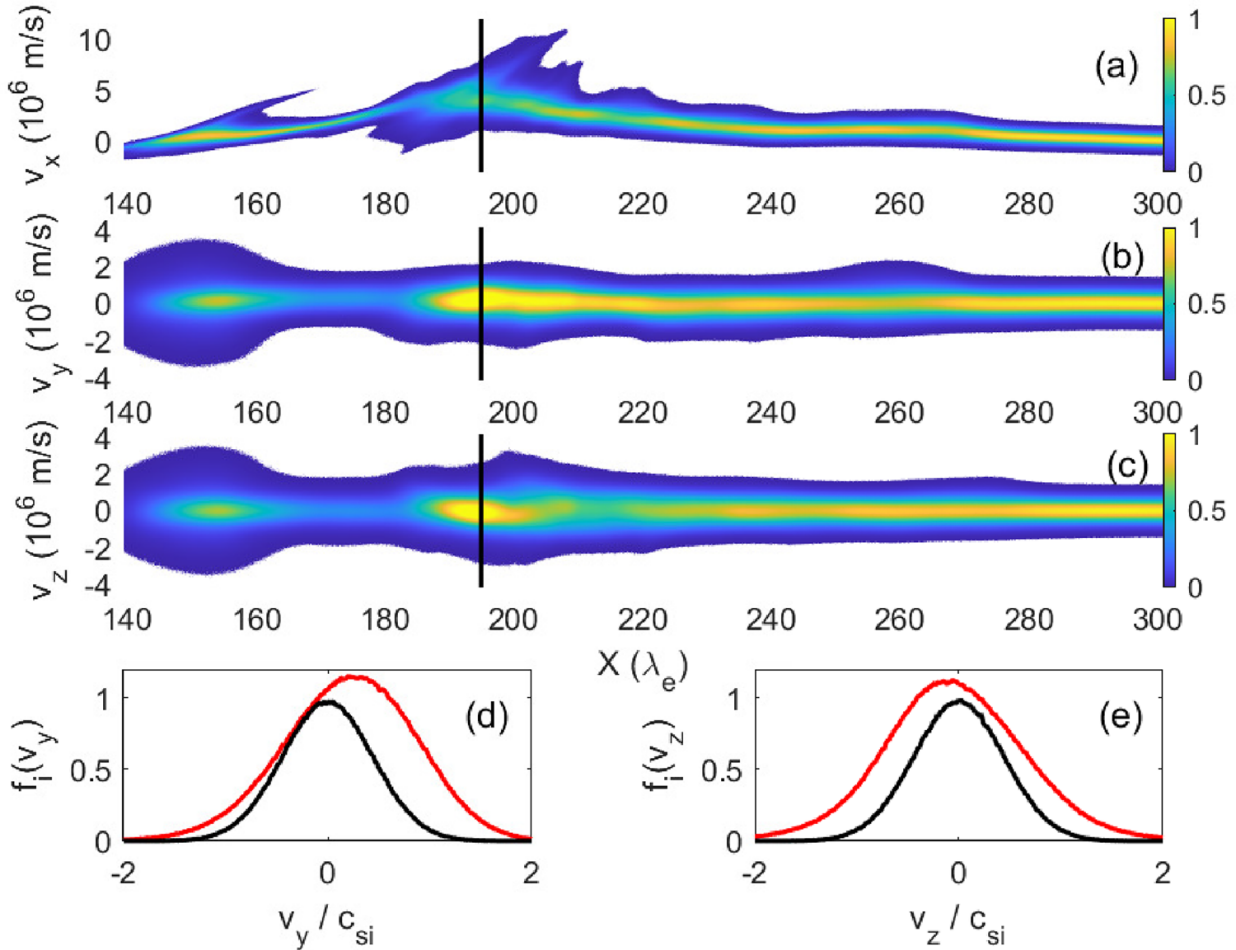
Box-averaged proton phase space densities $f_i$ at time $1614\omega _{pe}^{-1}$ normalized to the maximum at $t=0$. Panels (**a**–**c**) show the distributions $f_i(x,v_x)$, $f_i(x,v_y)$, and $f_i(x,v_z)$, respectively. The color scale is linear. The vertical lines denote $x=195\lambda _e$. Panels (**d**, **e**) compare $f_i(v_y)$ and $f_i(v_z)$ at $x=195\lambda _e$ (red) and $x=350\lambda _e$ (black), respectively.

Figure 8 shows projections of the box-averaged proton phase space density in the three planes formed by *x* and the velocity components $v_x$, $v_y$, and $v_z$ at time $t=1614\omega _{pe}^{-1}$.

Figure 8a reveals that the fastest protons reached a velocity $v_x\approx 10^7$m/s and that their mean velocity near $x=195\lambda _e$ exceeds $3 \times 10^6$m/s, which can explain their rapid motion along *x* in Figs. 7(b, d). Since Fig. 8 shows the box-average, we find that the magnetic flux tubes are effective in coupling the directed flow energy of the injected pair cloud to protons. The motion of the magnetic flux tubes along *y* allows them to accelerate protons in a wide spatial interval.

Figure 8b, c show that while the mean speed of protons along *y* and *z* is close to zero, the spread in the proton velocities varies along *x*. This spread can be caused by proton heating or by a spatially varying bulk motion in the y-z plane. Figure 7 evidences the latter.

Proton hot spots with particle speeds exceeding $c_{si}$ are found near $150\lambda _e$, $200\lambda _e$, and in Fig. 8b at $x=260\lambda _e$. Figure 8d, e compare the velocity distributions at $x=195\lambda _e$ and along $v_y$ and $v_z$ to the initial ones. The velocity distribution in Fig. 8d has its maximum near $0.3c_{si}$ or $3 \times 10^5$ m/s and the distribution $f_i(x=195\lambda _e,v_z)$ in Fig. 8e near $-0.1c_{si}$; the velocity distributions are thus not necessarily Maxwellians.

### Final time $t=t_{sim}$

**Fig. 9:**
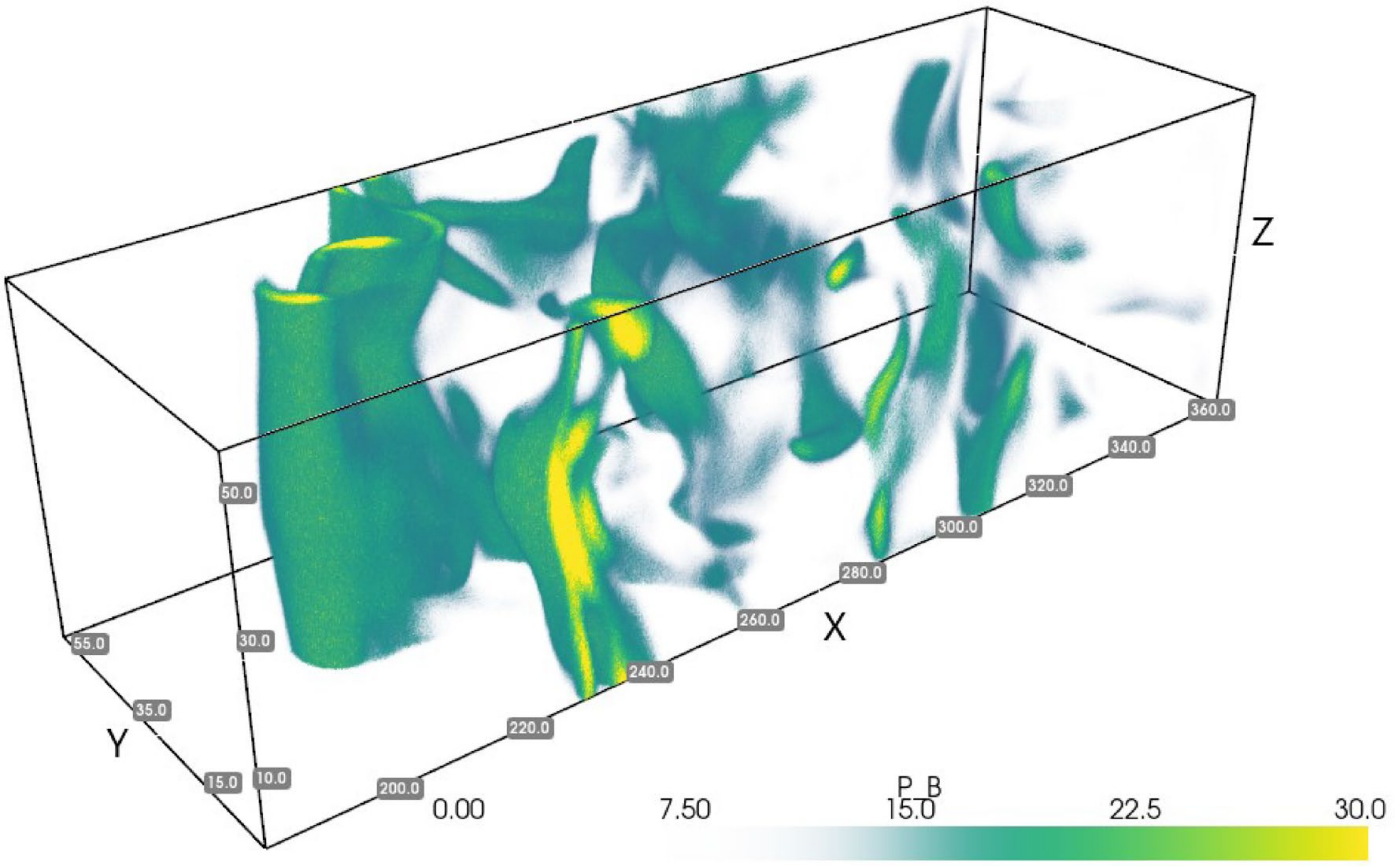
Magnetic pressure $P_B$ at time $t_{sim}$. The color and opacity scales are clamped to 30. Magnetic flux tubes are present with peak values of $P_B(x,y,z)$ comparable to 40. At this time the ep plasma shock and the filamentary magnetic field of its transition layer have left the simulation box.

Figure 9 shows $P_B(x,y,z)$ at time $t_{sim}$.

The magnetic flux tubes are spread over a larger *x*-interval compared to Fig. 5. Their peak pressure and alignment remain unchanged, underlining their stability. This is the case only for the magnetic flux tubes with $P_B(x,y,z) \ge 15$. Field lines with a lower amplitude, which are not visible in the selected color and opacity scale, are deformed and twisted by the momentum transfer from the pair plasma to the protons. The magnetic flux tubes at positions $x>280\lambda _e$ seem to vanish. The magnetic divergence law is fulfilled to round-off precision, but a divergence of field lines naturally leads to a decreasing magnetic pressure.

**Fig. 10:**
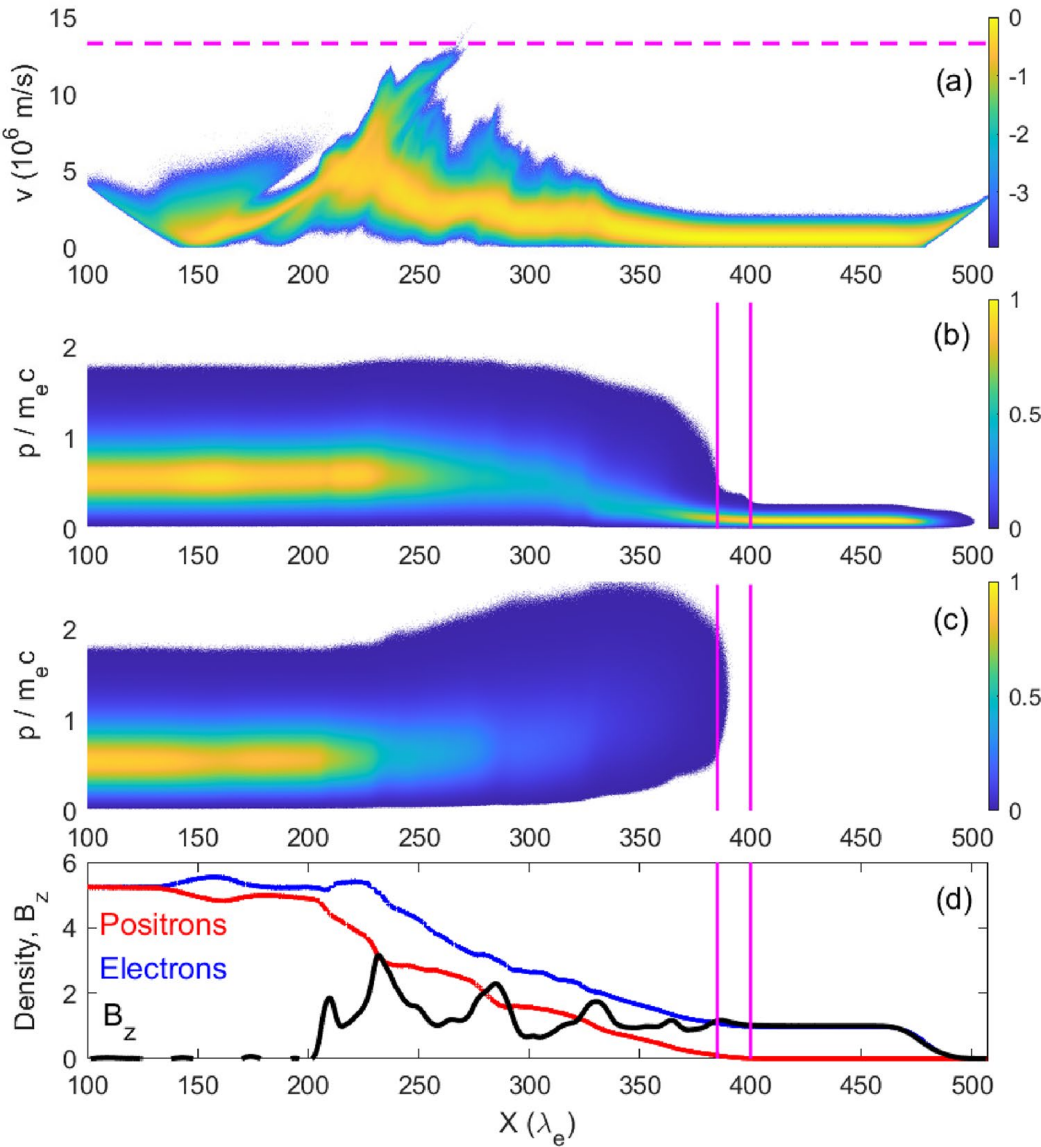
Plasma state at time $t=t_{sim}$. (**a**) 10-logarithmic phase space density of protons as a function of the velocity $v=|\textbf{v}|$. It is normalized to peak density at $t=0$. The dashed magenta line marks $v=13.3 \times 10^6$m/s. (**b**, **c**) Phase space densities of electrons and positrons on a linear scale, respectively. Both are normalized to the peak density of the electrons at $t=0$. (**d**) Box-averaged densities of electrons and positrons and the box-averaged magnetic $B_z$ component normalized to $B_0$. The vertical magenta lines in (**b**–**d**) mark $x=385\lambda _e$ and $400\lambda _e$.

Figure 10 shows the box-averaged velocity distributions of the three plasma species at time $t_{sim}$. Movie 2 animates these distributions in time.

The protons in Fig. 10a are accelerated over a broad spatial interval, which coincides with the transition layer of the magnetic field. The fastest protons reach a velocity $\approx 13.3 \times 10^6$ m/s. This speed is about $14c_{si}$ or 12$v_{fm}$.

Figure 10b, c show practically identical distributions of electrons and positrons in the interval $x<230\lambda _e$. A curve fit showed that they match the distribution of a drifting Maxwellian with mean speed 0.35*c* along *x* and temperature 11$T_0$. Both distributions start to diverge at $x>230\lambda _e$. The peak momentum of electrons decreases with increasing *x*, while that of positrons increases.

The momentum transfer from the electrons to the positrons is electrostatic in nature. In order to let the electric field vanish, the electric current of the moving electrons must be compensated by the electric current of two positively charged species with different masses, which move in the same direction and have a total charge equal to that of the electrons.

Protons are not easily accelerated to the electron’s mean speed. An electric field grows, which reduces the electron speed at large *x* and accelerates protons and positrons in the expansion direction of the electrons. The electrons have lost most of their energy at $x\approx 385\lambda _e$.

A heated electron population is observed in the interval $385\lambda _e< x < 400\lambda _e$, where hardly any positrons are present. The box-averaged $B_z$ component starts to increase in this interval. This, together with the electron momentum distribution in Fig. 10(b) and the absence of positrons between the magenta lines, suggests that electrons drift in the magnetic field and amplify it.

We note that the presence of magnetic flux tubes implies that the front of the expanding ep plasma is not necessarily planar along *y* and *z*, which is not taken into account by the box averaging.

## Discussion

We modeled the collision between an unmagnetized electron-positron (ep) plasma with the mean speed $\textbf{v}_0=(v_0,0,0)$ and $v_0=0.6c$ and an electron–proton (ei) plasma at rest. The ei plasma filled an interval with a finite length along *x* and was permeated by a uniform magnetic field with a direction perpendicular to $\textbf{v}_0$.

Electrons and positrons were continuously injected at one simulation boundary to maintain the density of the expanding ep plasma. The injected electrons and positrons had the same density, temperature, and mean velocity as the ep plasma that was initially present in the simulation box.

The purpose of our 3D PIC simulation was to identify the key processes which develop when an unmagnetized ep plasma collides with a magnetized ei plasma and their characteristic spatial and time scales. We identified the scales for the case of matching densities between the ep plasma and the ei plasma and for an electron plasma frequency $\omega _{pe}$ that exceeded the electron gyro-frequency $\omega _{ce}$ by the factor 11 in the ei plasma.

We obtained the following results. The diamagnetic current of the ep plasma drifting in the magnetic field of the ei plasma pushed it in the direction of $\textbf{v}_0$. Charge density waves grew in the drifting ep plasma and destabilized the initially planar magnetic boundary on a time scale $\sim 2\pi \omega _{ce}^{-1}$ turning it into magnetic flux tubes.

The most powerful magnetic flux tubes had a magnetic pressure that exceeded the initial thermal pressure of the electron–proton plasma by 1-2 orders of magnitude. Even if we take into account that the electrons increased their temperature by an order of magnitude during their interaction with the magnetized ei plasma, equi-partition between magnetic and electron thermal energy is still maintained within the magnetic flux tube.

The thermal gyro-radius $v_{te}/\omega _{ce}$ of electrons with temperature $T_e$ and magnetic field amplitude $B_0$ is about $\lambda _e$. The scale over which the magnetic field is strong is thus comparable to the electron thermal gyro-radius and electrons will emit synchrotron jitter radiation^14^.

The magnetized ei plasma formed an obstacle to the ep plasma. A collisionless shock formed in the ep plasma near the discontinuity that separated them from the ei plasma. The shock detached from the discontinuity within $20\pi \omega _{ce}^{-1}$ and propagated upstream. In the rest frame of the injected ep plasma, the shock was 4 times faster than the sound speed $c_{se}$. It compressed the inflowing upstream ep plasma by a factor 4, creating a hot and dense cocoon between the shock and the ei plasma. Its large thermal pressure let the ep plasma of the cocoon slowly expand into the ei plasma.

The ram pressure of the ep plasma accelerated the ei plasma by electrostatic and magnetic forces. The electrostatic force arises because of the proton inertia. Protons cannot easily keep up with electrons. An electrostatic field grows, which slows down electrons and accelerates positrons and protons. The magnetic forces were mediated by the magnetic flux tubes. The magnetic field amplitude was high enough to trap electrons and push them across the protons inducing an electric field.

Within $1600\omega _{pe}$ or $145\omega _{ce}^{-1}$ , the electromagnetic force accelerated protons up to 10 times the fast magnetosonic speed $v_{fm}$. At the final simulation fime $3100\omega _{pe}^{-1}$ or $375\omega _{ce}$, their peak speed was not much higher suggesting that the protons had reached their peak velocity for the selected plasma configuration. The proton’s mean velocity along *x* exceeded 3$v_{fm}$ near the magnetic flux tubes. The bulk speed of the protons relative to $v_{fm}$ is supersonic. This is not necessarily the case within the local plasma. The presence of positrons and the large temperature of the heated ep plasma, together with the larger Alfvén speed in the amplified magnetic field, increases the local fast magnetosonic speed. Indeed, no steepening of the proton front into a shock was observed. Movie 2 showed the onset of wave breaking. The accelerated protons were slower than the ep plasma wave front and they can thus not catch up with it resembling the proton acceleration by an expanding pair plasma^15^.

Although the drift instability, which was responsible for the formation of the magnetic flux tubes, led to waves with a wavelength of 3$\lambda _e$ on short time scales, the study of the collision boundary will remain out of reach for laboratory studies for some time. The size of the discontinuity perpendicular to the collision direction and the magnetic field direction as well as the long evolution times require a number of ep pairs that is well outside range for what contemporary lasers can create.

The injection of pair plasma resulted in two artifacts at late times. The injected beam of pair plasma developed velocity oscillations when the pair shock approached the boundary. Such oscillations tend to occur in PIC simulations when an unequal number of electrons and positrons leave an open boundary. The net current near the boundary induces an electric field, which causes an instability of the injected pair plasma beam. These oscillations were absent early in the simulation, implying that the formation and early evolution of the pair plasma shock were captured correctly. The shock speed and the plasma compression did not change at late times, suggesting that it is robust against velocity oscillations in the inflowing pair plasma beam.

The second artifact was an accumulation of pair plasma near the injector, which propagated into the box. The injected beam and the pair plasma, which is already in the simulation box, react via beam instabilities. Such beam instabilities thermalize the pair plasma distribution and the simulation showed that the pair plasma was quasi-thermal when it arrived at the transition layer separating it from the electron–proton plasma. The main effect of this artifact is thus a moderate increase of the pair plasma density and, thus, thermal pressure. An even larger pair plasma density in Ref.^8^ led to a qualitatively similar evolution of the magnetic flux ropes suggesting that they are robust against such changes. This robustness will be tested in future simulations.

## Supplementary Information

Supplementary Information 1.

Supplementary Information 2.

## Data Availability

The data that support the findings of this study are available from the corresponding author upon reasonable request.
